# Breastfeeding and childhood obesity: A 12‐country study

**DOI:** 10.1111/mcn.12984

**Published:** 2020-03-05

**Authors:** Jian Ma, Yijuan Qiao, Pei Zhao, Wei Li, Peter T. Katzmarzyk, Jean‐Philippe Chaput, Mikael Fogelholm, Rebecca Kuriyan, Estelle V. Lambert, Carol Maher, Jose Maia, Victor Matsudo, Timothy Olds, Vincent Onywera, Olga L. Sarmiento, Martyn Standage, Mark S. Tremblay, Catrine Tudor‐Locke, Gang Hu, for the ISCOLE Research Group

**Affiliations:** ^1^ Tianjin Women's and Children's Health Center Tianjin China; ^2^ Pennington Biomedical Research Center Baton Rouge Louisiana USA; ^3^ Children's Hospital of Eastern Ontario Research Institute Ottawa Ontario Canada; ^4^ Department of Food and Nutrition University of Helsinki Helsinki Finland; ^5^ St. Johns Research Institute Bangalore India; ^6^ Division of Exercise Science and Sports Medicine, Department of Human Biology University of Cape Town Cape Town South Africa; ^7^ Alliance for Research In Exercise Nutrition and Activity (ARENA), School of Health Sciences University of South Australia Adelaide South Australia Australia; ^8^ CIFI2D, Faculdade de Desporto University of Porto Porto Portugal; ^9^ Center of Studies from the Physical Fitness Research Laboratory de São Caetano do Sul, Sao Paulo Brazil; ^10^ Department of Recreation Management and Exercise Science Kenyatta University Nairobi Kenya; ^11^ School of Medicine Universidad de los Andes Bogota Colombia; ^12^ Centre for Motivation and Health Behaviour Change, Department for Health University of Bath Bath UK; ^13^ Department of Kinesiology University of Massachusetts Amherst Amherst Massachusetts USA

**Keywords:** breastfeeding, central, children, epidemiology, multination, obesity, obesitybody fat

## Abstract

This study aimed to examine the association between breastfeeding and childhood obesity. A multinational cross‐sectional study of 4,740 children aged 9–11 years was conducted from 12 countries. Infant breastfeeding was recalled by parents or legal guardians. Height, weight, waist circumference, and body fat were obtained using standardized methods. The overall prevalence of obesity, central obesity, and high body fat were 12.3%, 9.9%, and 8.1%, respectively. After adjustment for maternal age at delivery, body mass index (BMI), highest maternal education, history of gestational diabetes, gestational age, and child's age, sex, birth weight, unhealthy diet pattern scores, moderate‐to‐vigorous physical activity, sleeping, and sedentary time, exclusive breastfeeding was associated with lower odds of obesity (odds ratio [OR] 0.76, 95% confidence interval, CI [0.57, 1.00]) and high body fat (OR 0.60, 95% CI [0.43, 0.84]) compared with exclusive formula feeding. The multivariable‐adjusted ORs based on different breastfeeding durations (none, 1–6, 6–12, and > 12 months) were 1.00, 0.74, 0.70, and 0.60 for obesity (*P*_trend_ = .020) and 1.00, 0.64, 047, and 0.64 for high body fat (*P*_trend_ = .012), respectively. These associations were no longer significant after adjustment for maternal BMI. Breastfeeding may be a protective factor for obesity and high body fat in 9‐ to 11‐year‐old children from 12 countries.

**Key messages**

- Childhood obesity is potentially affected by many factors. Several studies have shown that breastfeeding has a significant protective effect on childhood obesity, whereas others have shown a weak effect or no effect.
- The present study examined the association between breastfeeding and the odds of childhood obesity in 9‐ to 11‐year‐old children from 12 countries.
- We found that breastfeeding is associated with significantly reduced odds of general obesity and high body fat in 9‐ to 11‐year‐old children from 12 diverse countries.

## 1 INTRODUCTION

Obesity is an important lifestyle‐related public health problem worldwide. The prevalence of obesity in children has risen dramatically during the past few decades not only in developed countries but also in developing countries (Wu, 2013). Indeed, one recent review has reported that the prevalence of childhood overweight and obesity rose by 47.1% between 1980 and 2013 worldwide (Ng et al., 2014). Childhood overweight is a strong predictor of adult obesity (Whitaker, Wright, Pepe, Seidel, & Dietz, 1997) and other adverse health consequences, especially type 2 diabetes and cardiovascular disease in adolescence and adult life (Daniels, 2009; Goran, Ball, & Cruz, 2003). Thus obesity prevention is key to controlling its epidemic and identification of modifiable risk and protective factors is essential.

The benefits of breastfeeding in early childhood are well established. Breastfeeding is the recommended form of nutrition for the first 6 months of infant life. Current data on the impact of breastfeeding on overweight in childhood provide equivocal findings. Some studies have shown a significant protective effect (Armstrong & Reilly, 2002; Gillman et al., 2001; Grummer‐Strawn & Mei, 2004; Rito et al., 2019), whereas others have shown a weak effect or no effect (Hediger, Overpeck, Kuczmarski, & Ruan, 2001; Victora, Barros, Lima, Horta, & Wells, 2003). Data from two recent meta‐analyses have shown that breastfeeding was associated with a significantly reduced risk of later obesity in children (Horta, Loret de Mola, & Victora, 2015; Yan, Liu, Zhu, Huang, & Wang, 2014). The inconsistent nature of results from past work suggests that the association between breastfeeding and childhood overweight may be modified by one or more extraneous variables. Obesity is a multifactorial disorder with genetic, socio‐economic status, and lifestyle factors (e.g., physical activity and eating habits) as important predisposing factors (Hossain, Kawar, & El Nahas, 2007). Moreover, maternal history of gestational diabetes, birth weight, children's moderate‐to‐vigorous physical activity (MVPA), diet, sedentary behaviours, and sleeping duration may confound the association between breastfeeding and the risk of later childhood obesity. However, few studies were able to adjust for these factors simultaneously. The aim of the present study was to examine the association between breastfeeding and the odds of obesity in 9‐ to 11‐year‐old children from 12 countries while controlling for these purported confounders.

## 2 METHODS

### 2.1 Study design

The International Study of Childhood Obesity, Lifestyle and the Environment (ISCOLE) is a multinational cross‐sectional study conducted at urban and suburban sites in 12 countries (Australia, Brazil, Canada, China, Colombia, Finland, India, Kenya, Portugal, South Africa, United Kingdom, and the United States; Katzmarzyk et al., 2013). These countries were selected to represent diverse geographic and income groups according to the World Bank Classification (Table 1). More details on the study design and methods can be found elsewhere (Katzmarzyk et al., 2013). Written informed consent was obtained from parents or legal guardians, and child assent was also obtained as required by local Institutional/Ethical Review Boards before participation in the study.

**Table 1:** International study of childhood obesity, lifestyle and the environment (ISCOLE) field site characteristics

Country	World bank classification	No. of study samples		
		Boys	Girls	Total
Australia	High income	182	204	386
Canada	High income	192	251	443
Finland	High income	190	211	401
Portugal	High income	221	312	533
United Kingdom	High income	141	183	324
United States	High income	150	213	363
Brazil	Upper middle income	171	183	354
China	Upper middle income	221	192	413
Colombia	Upper middle income	350	350	700
South Africa	Upper middle income	50	70	120
India	Lower middle income	185	229	414
Kenya	Low income	133	156	289
Total		2,186	2,554	4,740

### 2.2 Participants

A total of 7,372 children aged 9–11 years participated in the ISCOLE study, of whom 4,740 remained in the analytical sample for the present study after excluding participants who did not have valid data/information for accelerometry (*N* = 1,214), body mass index (BMI; *N* = 5), waist circumference (*N* = 5), percentage of body fat (*N* = 64), infant breast feeding (*N* = 426), birth weight (*N* = 355), gestational age (*N* = 108), maternal current BMI (*N* = 347), or other information (highest parental education, maternal history of gestational diabetes, and diet scores; *N* = 108). Participants who were excluded from the present analysis did not differ in BMI‐for‐age z‐scores but had a higher proportion of boys than those who were included in the analysis. Data were collected from September 2011 to December 2013.

### 2.3 Measurements

A demographic and family health history questionnaire was completed by parents or legal guardians. The questionnaire collected information on maternal highest education, maternal history of gestational diabetes, child's age, sex, birth weight, infant feeding mode, maternal age at delivery, and gestational age. The maternal highest education was collapsed into three categories: did not complete high school, completed high school or college, and completed bachelor or postgraduate degree. The maternal height and weight were collected in 9‐ to 11‐year‐old children. The child's parents or guardians were asked whether the child was fed breast milk, the age when the child completely stopped being fed breast milk, the age when the child was first fed formula, and the age when the child completely stopped fed formula. These responses were classified into three categories for the first 6 months: exclusive breastfeeding, mixed feeding, and exclusive formula feeding.

### 2.4 Dietary intake

A food frequency questionnaire that was adapted from the Health Behavior in School‐aged Children Survey (Currie et al., 2008; Mikkilä et al., 2015) was administered to all ISCOLE participants. The food frequency questionnaire asks the participants their “usual” consumption of 23 food categories, with response categories including never, less than once per week, once per week, 2–4 days per week, 5–6 days per week, once a day every day, and more than once a day. Two diet scores that represented an “unhealthy diet pattern” (with positive loadings for fast food, hamburgers, soft drinks, sweets, fried food, etc.) and a “healthy diet pattern” (with positive loadings for vegetables, fruit, whole grains, low‐fat milk, etc.) were obtained using principal components analyses (Mikkilä et al., 2015).

### 2.5 Anthropometry measurement

A battery of anthropometric measurements was taken according to standardized procedures across all study sites. Height was measured without shoes using a Seca 213 portable stadiometer (Hamburg, Germany), after a deep inhalation with the participant's head in the Frankfurt plane. Waist circumference was measured with a nonelastic tape held midway between the lower rib margin and the iliac crest at the end of a gentle expiration. Waist circumference was measured on the bare skin in all countries except in Australia where it was measured over light clothing. The regression equation (*y* = 0.994*x* − 0.42) developed by McCarthy et al. was applied to the Australian data to correct for the over‐the‐clothes measurement (McCarthy, Ellis, & Cole, 2003). Each measurement was repeated, and the average was used for analyses (a third measurement was obtained if the first two measurements were greater than 0.5 cm apart, and the average of the two closest measurements was used in analyses).

The participant's weight and body fat were measured using a portable Tanita SC‐240 Body Composition Analyser (Arlington Heights, IL, USA) after all outer clothing, heavy pocket items, and shoes and socks were removed. Two measurements were obtained, and the average was used in analyses (a third measurement was obtained if the first two measurements were more than 0.5 kg or 2.0% apart, for weight and percentage body fat, respectively, and the closest two were averaged for analyses). The Tanita SC‐240 showed acceptable accuracy for estimating percent body fat when compared with dual‐energy X‐ray absorptiometry, supporting its use in field studies (Barreira, Staiano, & Katzmarzyk, 2013). BMI was calculated by dividing weight in kilograms by the square of height in metres. BMI z‐scores were computed using age‐ and sex‐specific reference data from the World Health Organization (De Onis et al., 2007). General obesity was defined as BMI z‐scores greater than +2 SD. Central obesity was defined as waist circumference ≥ 90th percentile of National Health and Nutrition Examination Survey III reference (Fernandez, Redden, Pietrobelli, & Allison, 2004; Singh, 2006). High body fat was defined as body fat ≥90th percentile of National Health and Nutrition Examination Survey IV reference (Laurson, Eisenmann, & Welk, 2011).

### 2.6 Accelerometry

An ActiGraph GT3X+ accelerometer (ActiGraph, LLC, Pensacola, FL, USA) was used to objectively measure MVPA, sedentary time, and sleep period time. The accelerometer was worn at the waist on an elasticized belt on the right mid‐axillary line. Participants were encouraged to wear the accelerometer 24 hr per day (removing only for water‐related activities) for at least 7 days (plus an initial familiarization day and the morning of the final day), including two weekend days. The minimal amount of accelerometer data that was considered acceptable was 4 days with at least 10 hr of waking wear time per day, including at least one weekend day (Katzmarzyk et al., 2013). Nocturnal sleep duration was estimated from the accelerometer data using a fully automated algorithm for 24‐hr waist‐worn accelerometers that was validated for ISCOLE (Barreira et al., 2015). This algorithm produces more precise estimates of sleep duration than previous algorithms and captures total sleep time from sleep onset to the end of sleep, including all epochs and wakefulness after onset (Barreira et al., 2015). The weekly total sleep time averages were calculated using only days where valid sleep was accumulated (total sleep period time ≥ 160 min) and only for participants with at least three nights of valid sleep, including one weekend day (Tudor‐Locke, Barreira, Schuna, Mire, & Katzmarzyk, 2014). After exclusion of total sleep time and awake nonwear time (any sequence of ≥20 consecutive minutes of zero activity counts), MVPA was defined as all activity ≥574 counts per 15 s and total sedentary time (SED) as all epochs ≤25 counts per 15 s, consistent with the widely used Evenson cut‐offs (Evenson, Catellier, Gill, Ondrak, & McMurray, 2008).

### 2.7 Statistical analyses

One‐way analysis of variance and chi‐square test were used to compare mean levels of continuous variables and percentage of categorical variables among children with different feeding mode status. Multilevel logistic regression models were used to estimate the association between infant feeding mode and the odds of childhood obesity, central obesity, and high body fat. We defined child as Level 1, school as Level 2, and study site as Level 3 for the multilevel analyses. Study site and school were considered to have random effects. The denominator degrees of freedom for statistical tests pertaining to fixed effects were calculated using the Kenward and Roger (1997) approximation. The analyses were adjusted for maternal age at delivery, current maternal BMI, maternal education, maternal history of gestational diabetes, birth weight, child's unhealthy diet pattern scores, MVPA, sleeping duration, sedentary behaviours time, and child's age and sex. The criterion for statistical significance was *P* < .05. All statistical analyses were performed with SPSS for Windows, Version 21.0 (Statistics 21, SPSS, IBM, USA) or SAS for Windows, Version 9.4 (SAS Institute, Cary, NC, USA).

## 3 RESULTS

A total of 4,740 children (2,186 boys and 2,554 girls) were included in the present study. The distribution of sample sizes across sites is presented in Table 1. General characteristics of the study population are presented in Table 2. The overall prevalence of general obesity, central obesity, and high body fat were 12.3%, 9.9%, and 8.1%.

**Table 2:** Characteristics of study participant by different feeding mode at 6 months

Characteristic	Exclusive breastfeeding	Mixed feeding	Exclusive formula feeding	Total	*P* value
Maternal characteristics					
Age at delivery (years)	28.5 (5.9)	28.6 (5.6)	27.7 (5.7)	28.4 (5.7)	.003
Current body mass index (kg/m^2^)	25.2 (4.5)	25.6 (4.7)	27.1 (6.5)	25.6 (4.9)	<.001
History of gestational diabetes, *N* (%)	63 (3.5)	108 (4.7)	35 (5.3)	206 (4.3)	.082
Education, *N* (%)					<.001
Did not complete high school	473 (26.5)	405 (17.7)	171 (25.8)	1,049 (22.1)	
Completed high school/some college	786 (44.0)	1,007 (43.9)	360 (54.3)	2,153 (45.4)	
Bachelor's degree or postgraduate degree	526 (29.5)	880 (38.4)	132 (19.9)	1,538 (32.4)	
Offspring characteristics at birth					
Sex, *N* (%)					.064
Boys	850 (47.6)	1,017 (44.4)	319 (48.1)	2,186 (46.1)	
Girls	935 (52.4)	1,275 (55.6)	344 (51.9)	2,554 (53.9)	
Birth weight (g)	3310 (566)	3,259 (579)	3274 (609)	3,280 (579)	.020
Gestational age (weeks)	38.8 (2.0)	38.6 (2.2)	38.3 (2.4)	38.6 (2.2)	<.001
Offspring characteristics at age 9–11 years					
Age (years)	10.4 (0.6)	10.4 (0.5)	10.4 (0.6)	10.4 (0.6)	.031
Body mass index (kg/m^2^)	18.2 (3.4)	18.3 (3.3)	19.1 (3.8)	18.4 (3.4)	<.001
Waist circumference (cm)	64.0 (8.8)	64.3 (8.6)	65.1 (9.7)	64.3 (8.9)	.033
Body fat (%)	20.3 (7.5)	20.8 (7.5)	22.3 (8.1)	20.8 (7.6)	<.001
Unhealthy diet pattern score	−0.2 (0.7)	−0.2 (0.8)	0.1 (1.1)	−0.1 (0.9)	<.001
Moderate‐to‐vigorous physical activity (min/day)	60.3 (24.5)	59.9 (25.2)	57.0 (23.0)	59.6(24.7)	.012
Sedentary time (min/day)	519 (67.6)	516 (68.1)	522 (67.3)	518 (67.8)	.126
Duration of night sleep (min/day)	528 (53.0)	527 (53.2)	531 (53.6)	528 (53.2)	.168
General obesity, *N* (%)^a^	204 (11.4)	261 (11.4)	119 (17.9)	584 (12.3)	<.001
Central obesity, *N* (%)^b^	173 (9.7)	215 (9.4)	82 (12.4)	470 (9.9)	<.071
High body fat, *N* (%)^c^	120 (6.7)	175 (7.6)	89 (13.4)	384 (8.1)	<.001

Data are means (*SD*) or number (%).

a General obesity was defined as BMI z‐score ≥ 2 SD for age and gender specific distribution based on the World Health Organization growth reference.

b Central obesity was defined as waist circumference ≥ 90th percentile for age and gender specific distribution using National Health and Nutrition Examination Survey III reference.

c High body fat was defined as body fat ≥90th percentile for age and gender specific distribution using National Health and Nutrition Examination Survey IV reference.

After adjustment for maternal age at delivery, education, history of gestational diabetes, gestational age, and child's age, sex, birth weight, unhealthy diet pattern scores, MVPA, sleeping time, and SED (multivariable‐adjusted Model 2), the odds ratio (OR) of childhood general obesity was significantly lower among children with exclusive breastfeeding (OR 0.66, 95% confidence interval, CI [0.50, 0.88]) compared with those with exclusive formula feeding (reference group), and this association was still significant after additional adjustment for current maternal BMI (multivariable‐adjusted Model 3; OR 0.76, 95% CI [0.57, 1.00]; Table 3). We did not find any significant associations of exclusive breastfeeding, mixed feeding, and exclusive formula feeding with the odds of central obesity in different multivariable‐adjusted models (Table 4). The multivariable‐adjusted (Model 3) ORs of high body fat were significantly lower among children with exclusive breastfeeding (OR 0.60, 95% CI [0.43, 0.84]) and among children with mixed feeding (OR 0.72, 95% CI [0.52, 0.98]) compared with those with exclusive formula feeding (Table 5).

**Table 3:** Odds ratios of obesity at 9‐ to 11‐year‐old children by different feeding mode and breastfeeding duration

	No. of participates	No. of cases	Odds ratios (95% confidence intervals)		
			Model 1^a^	Model 2^b^	Model 3^c^
Feeding mode					
Exclusive formula feeding	663	119	1.00	1.00	1.00
Mixed feeding	2,292	261	0.73 [0.57, 0.95]	0.75 [0.57, 0.97]	0.83 [0.64, 1.09]
Exclusive breastfeeding	1,785	204	0.70 [0.54, 0.91]	0.66 [0.50, 0.88]	0.76 [0.57, 1.00]
Breastfeeding duration (months)					
None	663	119	1.00	1.00	1.00
1–6	1359	152	0.74 [0.56, 0.98]	0.74 [0.56, 0.99]	0.84 [0.62, 1.12]
7–12	1379	159	0.70 [0.53, 0.92]	0.70 [0.52, 0.93]	0.80 [0.60, 1.08]
>12	1339	154	0.72 [0.54, 0.96]	0.68 [0.50, 0.91]	0.75 [0.55, 1.02]
*P* for trend			.046	.020	.083

a Model 1 adjusted for childs age and sex.

b Model 2 adjusted for maternal age at delivery and education, maternal history of gestational diabetes and gestational age, and childs unhealthy diet pattern scores, birth weight, moderate‐to‐vigorous physical activity, sleeping time, sedentary time, age, and sex.

c Model 3 adjusted for variables in Model 3 and also for maternal body mass index.

**Table 4:** Odds ratios of central obesity at 9‐ to 11‐year‐old children by different feeding mode and breastfeeding duration

	No. of participates	No. of cases	Odds ratios (95% confidence intervals)		
			Model 1^a^	Model 2^b^	Model 3^c^
Feeding mode					
Exclusive formula feeding	663	82	1.00	1.00	1.00
Mixed feeding	2,292	215	0.80 [0.60, 1.07]	0.85 [0.62, 1.13]	0.96 [0.71, 1.31]
Exclusive breastfeeding	1,785	173	0.81 [0.60, 1.09]	0.80 [0.58, 1.08]	0.92 [0.67, 1.27]
Breastfeeding duration (months)					
None	663	82	1.00	1.00	1.00
1–6	1,359	123	0.87 [0.64, 1.19]	0.89 [0.64, 1.22]	1.02 [0.73, 1.43]
7–12	1,379	127	0.73 [0.54, 1.00]	0.76 [0.55, 1.05]	0.90 [0.64, 1.25]
>12	1,339	138	0.83 [0.60, 1.14]	0.81 [0.58, 1.12]	0.91 [0.65, 1.27]
*P* for trend			.218	.173	.407

a Model 1 adjusted for childs age and sex.

b Model 2 adjusted for maternal age at delivery and education, maternal history of gestational diabetes and gestational age, and childs unhealthy diet pattern scores, birth weight, moderate‐to‐vigorous physical activity, sleeping time, sedentary time, age, and sex.

c Model 3 adjusted for variables in Model 3 and also for maternal body mass index.

**Table 5:** Odds ratios of high body fat at 9‐ to 11‐year‐old children by different feeding mode and breastfeeding duration

Outcomes	No. of participate	No. of cases	Odds ratios (95% confidence intervals)		
			Model 1^a^	Model 2^b^	Model 3^c^
Feeding mode					
Exclusive formula feeding	663	89	1.00	1.00	1.00
Mixed feeding	2,292	175	0.62 [0.46, 0.83]	0.63 [0.46, 0.85]	0.72 [0.52, 0.98]
Exclusive breastfeeding	1,785	120	0.56 [0.41, 0.76]	0.52 [0.38, 0.72]	0.60 [0.43, 0.84]
Breastfeeding duration (months)					
None	663	89	1.00	1.00	1.00
1–6	1,359	102	0.65 [0.47, 0.90]	0.64 [0.46, 0.89]	0.73 [0.52, 1.02]
7–12	1,379	84	0.48 [0.34, 0.66]	0.47 [0.33, 0.66]	0.55 [0.38, 0.79]
>12	1,339	109	0.68 [0.49, 0.95]	0.64 [0.46, 0.90]	0.72 [0.51, 1.02]
*P* for trend			.024	.012	.060

a Model 1 adjusted for childs age and sex.

b Model 2 adjusted for maternal age at delivery and education, maternal history of gestational diabetes and gestational age, and childs unhealthy diet pattern scores, birth weight, moderate‐to‐vigorous physical activity, sleeping time, sedentary time, age, and sex.

c Model 3 adjusted for variables in Model 3 and also for maternal body mass index.

The multivariable‐adjusted (Model 2) ORs based on different breastfeeding durations (none, 1–6, 6–12, and > 12 months) were 1.00, 0.74, 0.70, and 0.60 for obesity (*P* for trend = .020; Table 3) and 1.00, 0.64, 047, and 0.64 (*P* for trend = .012; Table 5), respectively. These associations were no longer significant for childhood obesity and were still significant for high body fat among children with breastfeeding at 7–12 months after additional adjustment for maternal BMI.

## 4 DISCUSSION

In this multinational cross‐sectional study, we found that breastfeeding was a protective factor for childhood general obesity and high body fat in 9‐ to 11‐year‐old children from 12 countries.

An increased prevalence of childhood overweight and obesity has been observed worldwide over the past few decades, indicating a need for strategies to prevent obesity. Therapeutic interventions aimed at encouraging weight loss in children with obesity are costly and have had unsatisfactory long‐term success rates (Canadian Medical Association, 1994). There is some evidence that the odds of obesity are primed by exposures early in life. Among these factors, breastfeeding has been hypothesized as a potential protective factor against overweight (Armstrong & Reilly, 2002; Gillman et al., 2001; Grummer‐Strawn & Mei, 2004; von Kries et al., 1999). Although numerous studies support the protective effect of increased breastfeeding duration against childhood and adolescent obesity, other studies do not. Vehapoglu et al. (2014) found no association between the duration of breastfeeding and childhood obesity in children aged 2–14 years. Our study with large sample sizes from 12 diverse countries found a stronger association of breastfeeding with the risk of high body fat, a significant association of breastfeeding with the risk of general obesity, and a nonsignificant association of breastfeeding with the risk of central obesity among children aged 9–11 years when potential confounders were controlled. Thus, our study suggested that previous studies with only BMI measure in children may have underestimated the true effect of breastfeeding on obesity risk. The lack of effect of breastfeeding on central adiposity risk was found, and more studies are needed to assess this association.

Childhood overweight and obesity reflect the convergence of many biological, economic, and social factors. No single factor has been shown to protect a child from obesity. The difference in the results of previous studies may be due to the control of different confounding factors. Inconsistent findings in previous research may be a consequence of several limitations such as varying definitions of breastfeeding, different age periods of measurement, and lack of adjustment for additional possible confounders. Breastfeeding from diabetic mothers may increase the risk of becoming overweight (Plagemann, Harder, Franke, & Kohlhoff, 2002). Increased glucose and insulin content of breast milk of diabetic mothers (Jovanovic‐Peterson, Fuhrmann, Hedden, Walker, & Peterson, 1989) may contribute to effects of breastfeeding on infant growth, although some investigators have found no difference in macronutrient content of breast milk of well‐controlled diabetic mothers (van Beusekom et al., 1993). A recent study showed that the effect of breastfeeding on reducing the risk of obesity in later years is achieved in the first year of life (Scholtens et al., 2007). Current dietary and lifestyle factors are maybe more responsible for reducing the risk of obesity. So the evaluation of physical activity and dietary intake of the children are important confounding factors in assessing the relationship between obesity and breastfeeding. Al‐Qaoud and Prakash found that maternal BMI was a strong predictor of child BMI status. Children of mothers with obesity are 1.94 times more likely to be overweight and 2.63 times more likely to be obese than children of healthy‐weight mothers. Many studies have also shown that maternal BMI was a strong predictor of obesity (Burdette, Whitaker, Hall, & Daniels, 2006; Hediger et al., 2001). Some studies found mixed effects of breastfeeding on a child's weight status, depending on the degree to which confounders were controlled. Our findings made it possible to adjust for several important confounding factors, such as maternal age at delivery, maternal education, maternal history of gestational diabetes, gestational age, current maternal BMI, child's age and sex, unhealthy diet pattern scores, healthy diet pattern scores, MVPA, sleep time, and sedentary time.

Our study provides good evidence that breastfeeding may be protective of the development of obesity in childhood. Several possible biological mechanisms could be responsible for the protection of breastfeeding against childhood obesity. First, the nutritional and bioactive characteristics of human milk might be associated with childhood obesity. Breast milk contains hormones such as leptin, adiponectin, and ghrelin, and all these factors relate to the regulation of the content of adipose tissue. Studies have found that formula‐fed children might have higher plasma concentrations of insulin compared with those who had breastfeeding, and these higher concentrations of insulin would be expected to stimulate fat deposition and the early development of adipocytes (Lucas et al., 1980). Furthermore, breast milk also contains bioactive factors that may modulate epidermal growth factor and tumour necrosis factor, both of which are known to inhibit adipocyte differentiation in vitro (Hauner, Rohrig, & Petruschke, 1995). Second, early infant nutrition is one of the most powerful environmental factors determining early growth and development. After the first 3–4 months of life, breast‐fed infants gain less weight than formula‐fed infants (Kramer et al., 2002). Gaining less weight in infancy predicts lower rates of obesity in childhood and into adulthood (Gillman, 2010). Nutritional intake and metabolism in the critical or sensitive period of life development may lead to “programmed” or “metabolic imprinting” and will exert long‐term and lifelong effects on body structure, function, and substance metabolism. Third, the establishment of self‐regulation of food intake in infancy is extremely important to nutritional balance in childhood and even adulthood. It has been proposed that infants are born with some ability to regulate their energy intake in response to internal appetite cues (Birch & Fisher, 1998). However, this innate ability might be disrupted by the type of milk (human vs. nonhuman) and by the feeding mode (breast vs. bottle; Bartok & Ventura, 2009). It is postulated that breast‐fed infants have the ability to self‐regulate their energy intake to match their energy needs (Dewey & Lonnerdal, 1986). The sucking strength of infants varies according to their hunger, and the secretion of breast milk varies with the infant's sucking stimulation. Therefore, breast‐fed children can automatically control the food intake according to their own requirement, whereas formula‐fed infants are passive. Because parents do not think milk should be left in the bottle, it may cause formula‐fed children to overeat milk. The control of caregivers in formula feeding could lead to infants' poor self‐regulation on the basis of internal cues of hunger and satiety. Overconsumption of food increases the risk of obesity. To a greater extent than bottle‐fed infants, infants who are nursing typically let their mothers know when they are full by coming off the breast, which could lead to better self‐regulation of energy intake as they grow (Li, Fein, & Grummer‐Strawn, 2010).

There are several strengths in the present study. First, we used a globally diverse sample (including 12 countries from different geographic regions and economic levels) to test our hypothesis, thus increasing the external validity of our findings. These populations include children living in different stages of nutritional status including population with the double burden of malnutrition. Second, childhood obesity reflects the convergence of many biological, economic, and social factors. Accordingly, we collected data on many factors associated with obesity to control for the impact of confounding factors. Nevertheless, there are several limitations in our study. First, the cross‐sectional design precludes us from making cause‐and‐effect inferences. Second, this is a retrospective study. Breastfeeding data were based on self‐report, and mothers may forget when they introduced formula that could be biased or inaccurate; however, one study found that maternal recall was a valid and reliable estimate of breastfeeding initiation and duration (Li, Scanlon, & Serdula, 2005). Third, we did not collect the information whether the parent introduced solids/liquids in addition to breast milk before 4/6 months, which did not meet the strict definition of exclusive breastfeeding. Fourth, maternally reported birth weights, gestational age, and other neonatal events may have been inaccurately recalled.

## 5 CONCLUSION

In conclusion, breastfeeding was associated with significantly reduced odds of general obesity and high body fat in 9‐ to 11‐year‐old children from around the world. Greater allocation of health care and community resources to promote and support breastfeeding may benefit children and adolescents by reducing their odds for overweight and obesity.

## CONFLICTS OF INTEREST

The authors reported no other potential conflicts of interest.

## CONTRIBUTIONS

PK, JC, MF, RK, EL, CM, JM, VM, TO, VO, OS, MS, MT, CT, and GH designed the research study, performed the research, and revised the manuscript. JM, YQ, PZ, and WL analysed the data. JM, YQ, and HG wrote the paper. All authors have read and approved the final manuscript.
